# Antidepressant effects of Kai-Xin-San in fluoxetine-resistant depression rats

**DOI:** 10.1590/1414-431X20176161

**Published:** 2017-08-17

**Authors:** X.Z. Dong, D.X. Wang, Y.P. Lu, S. Yuan, P. Liu, Y. Hu

**Affiliations:** 1 Department of Clinical Pharmacology, General Hospital of Chinese People's Liberation Army, Beijing, China; 2 Department of Chinese Medicine, Shanxi University of Traditional Chinese Medicine, Jinzhong, Shanxi, China

**Keywords:** Kai-Xin-San, Fluoxetine-resistant depression, Chronic unpredictable mild stress, Inflammatory factor, Anti-depression

## Abstract

This study aimed to investigate the antidepressant effect and the mechanism of action of Kai-Xin-San (KXS) in fluoxetine-resistant depressive (FRD) rats. Two hundred male Wistar rats weighing 200±10 g were exposed to chronic and unpredictable mild stresses (CUMS) for 4 weeks and given fluoxetine treatment simultaneously. The rats that did not show significant improvement in behavioral indexes were chosen as the FRD model rats. These rats were randomly divided into four groups: FRD model control; oral fluoxetine and aspirin; oral KXS at a dose of 338 mg·kg^–1^·day^–1^; and oral KXS at a dose of 676 mg·kg^–1^·day^–1^. Rats continued to be exposed to CUMS and underwent treatment once a day for 3 weeks, then cytokine (COX-2, IFN-γ, IL-1β, IL-2, IL-4, IL-6, IL-10, TGF-β, and TNF-α) levels in the hippocampus and serum, and organ coefficients were measured. Both doses of KXS improved the crossing and rearing frequencies, sucrose-preference index, and body weight in FRD rats. KXS at a dose of 338 mg·kg^–1^·day^–1^reduced COX-2, IL-2, IL-6, TNF-α levels, increased IL-10 level in the hippocampus, and reduced IL-2 and TNF-α levels in serum. KXS at a dose of 676 mg·kg^–1^·day^–1^reduced TNF-α level in the hippocampus, reduced IL-2 and TNF-α levels in serum, and increased IFN-γ and IL-10 levels in the hippocampus and serum. There were no significant differences in organ-coefficients of the spleen among and between groups. The results suggested that oral administration of KXS in FRD rats was effective in improving behavior disorders by influencing various inflammatory pathways.

## Introduction

Major depression is a common and sometimes fatal disorder that has a worldwide prevalence greater than 15%. It is estimated that major depressive disorder will be the second largest contributor to the global burden of disease by 2020. Despite considerable advances in the treatment of major depressive disorder in the past few years, treatment-resistant depression (TRD) remains a common condition that affects approximately 30% of this population (1). Therefore, identifying a potential drug that is effective in treating resistant depression with low toxicity is important.

Many studies have shown that increased plasma concentrations of interleukin (IL)-1, IL-6, and tumor necrosis factor (TNF)-α were found in major depression patients with a history of poorer response to antidepressants than in treatment-responsive patients (2–4). Similarly, patients with increased inflammatory activity before treatment have been reported to be less responsive to antidepressants (5–7). Given these findings, researchers hypothesized that inflammation may influence the effects of antidepressants. To support this hypothesis, the chronic unpredictable mild stress (CUMS) paradigm was tested by administering lipopolysaccharide (LPS) daily before the stressor. It was found that pretreatment with LPS, mimicking inflammation, had no significant effect on depression-related behaviors but attenuated the antidepressant action of fluoxetine significantly, suggesting that inflammation might play a role in the pathophysiology of antidepressant resistance (8).

Kai-Xin-San (KXS) (9) is a well-known formula that was first recorded in an ancient Chinese book: "Tai Ping Hui Min He Ji Ju Fang". KXS consists of Ginseng (*Panax ginseng* C.A. Meyer), hoelen (*Wolf Poria cocos*, Schw), polygala (*Polygala tenuifolia* Willd), and Acorus (*Acorus tatarinowii* Schott) in a ratio of 3:3:2:2. For thousands of years, it has been a renowned Chinese herbal formula for treating depression and ameliorating various learning and memory deficits, such as desolation, moodiness, and forgetfulness.

Our previous studies in mice models have indicated that KXS has antidepressant-like effects as demonstrated by the tail suspension test and forced swim test. It also significantly elevated the levels of central monoamine neurotransmitters, including 5-hydroxytryptamine, dopamine, and noradrenaline (10,11). Simultaneously, KXS could ameliorate chronic fatigue syndrome by promoting proliferation of splenocytes in mice and modulate the detrimental effects of cytokines (12). KXS exerts its antidepressant-like and nootropic effect in a CUMS model by modulating the hypothalamic-pituitary-adrenal axis, monoamine neurotransmitter levels, and cholinergic systems (13).

Based on the biological effects of KXS that have been explored previously, the current study aimed to assess its potential antidepressant action and its influence on inflammatory processes to identify potential mechanisms in fluoxetine-resistant depressive rats.

## Material and Methods

### Reagents and drugs

Fluoxetine was purchased from Eli Lilly and Company (USA). Aspirin was purchased from Bayer Medicines Company (Germany). ELISA kits for TNF-α, TGF-β, IFN-γ, COX-2, IL-1β, IL-2, IL-4, IL-6, and IL-10 were purchased from R&D Company (USA). KXS was purchased from the LvYe Medicinal Material Company (China). KXS was supplied in powder form, which was derived from a mixture of the aqueous extract as described previously (14).

### Animals

In total, 200 male Wistar rats weighing 200±10 g were obtained from the Animal Breeding Center of the PLA General Hospital (Beijing, China). All rats were kept in a temperature- (23±2°C) and humidity-controlled (60±10%) facility on a 12-h light/dark cycle with free access to food and water. All animal experimental protocols were approved by the Animal Experimentation Ethics Committee of General Hospital of Chinese PLA. All animal handling procedures were performed in compliance with the ‘Principles of Laboratory Animal Care’ and the Chinese legislation for the use and care of laboratory animals.

### Fluoxetine-resistant depression model and drug administration

Twelve rats were randomly assigned as the normal control group, housed undisturbed in 4 per cage without contact with the stressed animals. The remaining 188 rats were used to replicate the CUMS model following the established protocol (15). Rats received 4 weeks of stress stimulations, which consisted of high-speed agitation (10 min), immobilization (2 h), tilted cage (12 h), deprivation of food or water (24 h), continuous illumination (24 h), and forced swimming in ice water (5 min). Rats were randomly assigned one stimulation daily from 3:00–5:00 pm over 4 weeks, and the stressed rats were housed in individual cages to sustain the depressive state until the end of the experiment. Among the 188 rats, 12 were randomly assigned as depression model control (CUMS group), treated with CUMS stimulations only (administered water orally, with no fluoxetine), used for screening the FRD rats; the other 176 rats were administered fluoxetine (20 mg· kg^–1^·day^–1^, orally) for 4 weeks simultaneously. After 4 weeks, behavior tests (crossing frequency, rearing times, and sucrose preference) were performed. Among the 176 rats, the rats whose behavior index had no significant improvement compared with the CUMS rats and was significantly lower than that of normal rats were chosen as the FRD model. Ultimately, 48 rats met the criteria and were randomly divided into four groups. The rats were sequentially given CUMS stress and treated with water (n=12, FRD model group), fluoxetine (20 mg·kg^–1^·day^–1^) combined with aspirin (20 mg·kg^–1^·day^–1^) (n=12, Flu+Aspirin group), KXS at 338 mg·kg^–1^·day^–1^ (n=12, KXS-338 group), and KXS at 676 mg^–1^·kg^–1^·day^–1^(n=12, KXS-676 group) orally at 9:00-10:00 am for 3 weeks. Twelve rats in the normal control group were continuously administered water. The time interval between fluoxetine and aspirin in the Flu+Aspirin group was 30 min.

### Open-field test

The locomotor activity was assessed to detect immobility or changes in motor activity in the open-field test performed on days 0, 28, and 46 of the experiment. The open-field apparatus was a cubic open field arena measuring 80 cm in length, 80 cm in width, and 60 cm in height. The box floor was divided into 25 squares (5 squares long × 5 squares wide). Rats were placed individually into the center of the arena and allowed to explore freely for 5 min. The floor was wiped cleaned with 70% ethanol between tests. The number of square line crossings with all four paws and rearing (when the rat stood on its hind limbs) were recorded.

### Sucrose-preference test

The tests were performed on days 0, 28, and 46 of the experiment. Before the sucrose-preference test, rats were deprived of food and water for 24 h and then fed with two pre-weighted bottles containing 1% sucrose solution and water for 1 h. Intake was measured by weighing the bottles before and after each test. All tests were carried out in the home cage to minimize extraneous novelty and disturbance. The sucrose preference was calculated as sucrose intake/total water intake (sucrose intake+water intake). Anhedonia was defined as a reduction in sucrose preference relative to baseline levels.

### Enzyme-linked immunosorbent assay

After the experiment, rats were anesthetized with an intraperitoneal injection of 10% chloral hydrate (0.35 mL/100 g body weight). Blood samples were collected, and serum was separated from aliquots of blood samples to determine the levels of serum inflammatory cytokines. The whole brain was then quickly removed with scissors, and the hippocampus was isolated, frozen in liquid nitrogen, and stored at –80°C for further biochemical analysis. The levels of cytokines (COX-2, IFN-γ, IL-1β, IL-2, IL-4, IL-6, IL-10, TGF-β, and TNF-α) in the hippocampus and serum were measured using a paired antibody quantitative ELISA kit according to the manufacturer's instructions. The plates were measured using a microtiter plate reader (Perkin-Elmer, USA). Data are reported as ng/mL.

### Statistical analysis

Data are reported as means±SE. Differences between groups were analyzed by one-way ANOVA followed by Dunnett's test. Data were analyzed statistically using SPSS 17.0 software (USA). P values less than 0.05 were considered statistically significant.

## Results

### Fluoxetine-resistant depression model and behavior evaluation

Results of the open-field tests and sucrose-preference tests showed that before CUMS stress treatment (day 0), the number of line crossings and rearings, sucrose-preference index and body weights between groups had no significant differences. After 4 weeks, compared with normal control, the model rats, which were treated with CUMS stress and fluoxetine (day 28), showed a significant decrease in crossing frequency, rearing frequency, sucrose-preference index, and body weight (P<0.05). After 3 weeks of treatment (day 46), compared with the model control (treated with water), rats treated with CUMS stress, and fluoxetine+aspirin showed a significant increase in crossing frequency and sucrose-preference index. Both doses of KXS reverted the reduced crossing frequency, rearing frequency, sucrose-preference index and body weights in fluoxetine-resistant rats (p<0.05) (Table 1).

**Table 1.** Effects of Kai-Xin-San (KXS) on behaviors measured on days 0, 28 and 46, in fluoxetine-resistant depression (FRD) rats exposed to chronic and unpredictable mild stress model.

Index/Days	Normal	Model	Flu+Aspirin	KXS-338	KXS-676
Crossings					
D0	175.9±0.2	180.6±52.3	169.0±14.5	167.1±45.8	163.9±25.4
D28	160.9±20.1	107.3±27.9##	111.8±29.8##	106.5±22.2##	119.1±24.8##
D46	152.5±38.6	101.5±19.4##	128.1±28.6*	122.1±15.9*	135.0±28.2*
Rearings					
D0	29.1±9.7	26.8±7.4	25.8±6.2	24.4±5.5	22.0±4.6
D28	20.1±5.9	12.0±4.9##	11.4±5.9##	13.9±4.2##	15.0±2.6##
D46	14.5±5.0	8.9±3.5##	8.4±4.1	9.3±4.2	9.8±5.5*
Sucrose preference (mL)					
D0	86.2±17.3	89.6±15.2	83.2±16.9	81.9±10.5	86.4±12.4
D28	97.2±6.7	79.5±3.2##	75.6±7.9##	73.4±14.6##	78.8±6.6##
D46	98.2±5.4	72.2±13.1#	86.2±9.1*	87.5±11.1*	92.3±4.1**
Body weight (g)					
D0	250.1±8.7	241.2±7.4	235.3±10.4	235.4±11.2	242.5±11.9
D28	416.2±23.3	335.1±7.5##	339.9±12.1##	335.3±16.2##	331.5±16.3##
D46	446.5±22.7	351.9±11.1##	349.4±10.6	371.4±18.6*	390.4±22.4**

Data are reported as means±SD, n=12. Groups: FRD model control (Model), oral fluoxetine and aspirin (Flu+Aspirin), oral KXS at a dose of 338 mg/kg, and 676 mg/kg.

#P<0.05,

##P<0.01 compared to untreated Normal group.

*P<0.05,

**P<0.01 compared to Model control group.

### Cytokines in the hippocampus

**Figure 1.**
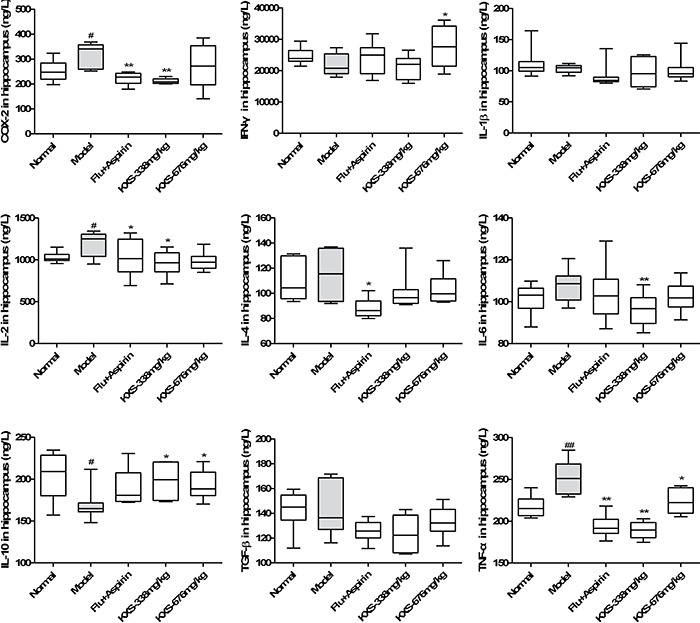
Effects of Kai-Xin-San (KXS) on cytokines in the hippocampus in fluoxetine-resistant depression (FRD) rats exposed to chronic and unpredictable mild stress model and randomly divided into four groups: FRD model control (Model), oral fluoxetine and aspirin (Flu+Aspirin), oral KXS at a dose of 338 mg/kg, and 676 mg/kg. Data are reported as means±SD, n=12. ^#^P<0.05, ^# #^P<0.01 compared to untreated Normal group. *P<0.05, **P<0.01 compared to Model control group (ANOVA followed by Dunnett's test).

ELISA experiments showed that levels of COX-2, IL-2, and TNF-α were increased significantly and levels of IL-10 were reduced in the hippocampus of fluoxetine-resistant rats, compared with normal rats (P<0.05). Fluoxetine in combination with aspirin decreased the levels of COX-2, IL-2, IL-4, and TNF-α in the hippocampus (p<0.05). KXS at a dose of 338 mg·kg^–1^·day^–1^ reduced levels of COX-2, IL-2, IL-6 and TNF-α, and increased IL-10 levels (P<0.05). KXS at a dose of 676 mg·kg^–1^·day^–1^ lowered TNF-α levels and increased IFN-γ levels in the hippocampus (p<0.05) (Figure 1).

### Cytokines in serum

**Figure 2.**
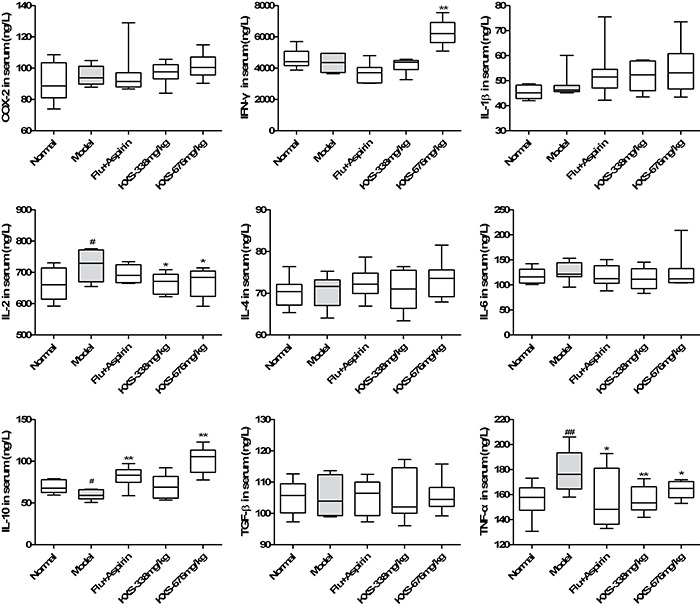
Effects of Kai-Xin-San (KXS) on cytokines in serum in fluoxetine-resistant depression (FRD) rats exposed to chronic and unpredictable mild stress model and randomly divided into four groups: FRD model control (Model), oral fluoxetine and aspirin (Flu+Aspirin), oral KXS at a dose of 338 mg/kg, and 676 mg/kg. Data are reported as means±SD, n=12. ^#^P<0.05, ^# #^P<0.01 compared to untreated Normal group. *P<0.05, **P<0.01 compared to Model group.

Compared with normal rats, levels of IL-2 and TNF-α were increased significantly, and the levels of IL-10 were reduced in the model group (Figure 2). This is similar to the results found in the hippocampus. Fluoxetine in combination with aspirin decreased TNF-α levels and increase IL-10 levels in serum. KXS at both doses reduced levels of IL-2 and TNF-α, KXS at a dose of 676 mg·kg^–1^·day^–1^ also increased IFN-γ and IL-10 levels in serum (P<0.05).

### Spleen coefficient

**Figure 3.**
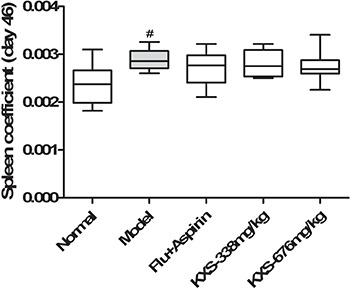
. Effects of Kai-Xin-San (KXS) on spleen coefficient in fluoxetine-resistant depression (FRD) rats exposed to chronic and unpredictable mild stress model and randomly divided into four groups: FRD model control (Model), oral fluoxetine and aspirin (Flu+Aspirin), oral KXS at a dose of 338 mg/kg, and 676 mg/kg. Data are reported as means±SD, n=12. ^#^P<0.05 compared to untreated Normal group.

Compared with that of normal rats, the spleen coefficient in fluoxetine-resistant rats was increased significantly (P<0.05), but treatment with fluoxetine in combination with aspirin or with KXS (both doses) had no significant influence on the spleen coefficient (Figure 3).

## Discussion

Depression is a psychological illness with high levels of disability and mortality, and is one of the most prevalent diseases in the world. In recent years, numerous studies have demonstrated a clear relationship between inflammation and the development of depression. For example, levels of IL-2, IL-6 in peripheral blood of patients with depression were significantly increased (16), levels of IL-2, IL-6, TNF-α in patients with first-episode depression were greater than that in normal patients (17), and overexpression of COX was found in the hippocampus of depressive rat models (18–20). These substances can inhibit the development of nerve cells, activate the hypothalamic-pituitary-adrenal axis simultaneously, increase the secretion of glucocorticoids and promote apoptosis (21). These findings suggest that changes in serum cytokine (such as COX, IL-2 and TNF-α) concentrations play an important role in the development and pathophysiology of depressive disorders. Depression is associated with cytokine secretion disorders, which are associated with immune activation (22,23). Drugs that can inhibit proinflammatory cytokines may produce antidepressant effects (24–26).

Furthermore, many studies have found that TRD is also accompanied by inflammatory dysregulation. Antidepressant-induced remission of depressive symptoms has also been associated with significant decreases in pro-inflammatory cytokine levels (27,28). Major depressive patients with a history of non-response to antidepressants were found to have increased plasma concentration of IL-1, IL-6 and acute phase reactants compared with treatment-responsive patients (29,30). Similarly, patients with increased inflammatory cytokines before treatment have been reported to be less responsive to antidepressant treatment (31,32). Aspirin is a non-selective COX inhibitor with a broad spectrum of pharmacological effects at multiple locations. Existing research shows that aspirin has antidepressant properties and accelerates antidepressant effects in preclinical models (33). Clinically, aspirin has been suggested to shorten the onset of action of selective reuptake inhibitors and to increase remission rates when added to fluoxetine in an open-label study of depressed patients previously non-responsive to fluoxetine alone (34). Therefore, it is essential to find effective treatments for TRD.

Traditional Chinese medicine in the treatment of depression is the focus of current research. It was documented that KXS could cure symptoms including desolation, moodiness, and forgetfulness, which are similar to symptoms of depression, such as depressed mood, anxiety, and impairment in learning and memory (13). As the principal herb of KXS, ginseng has been demonstrated to improve learning and memory in animals (10). 3,6′-Disinapoyl sucrose is an active oligosaccharide ester found in *Polygala tenuifolia* Willd, exhibits notable antidepressant effects in pharmacological depression models, and alleviates stress-induced behavioral abnormalities (15,35).

CUMS model is accepted as a valuable method for inducing experimental depression in rats. At least 20–30% of depressive rats do not respond to fluoxetine treatment (36). Aspirin can assist the treatment of depression through its anti-inflammatory effects, which led us to verify if KXS also played a role in regulating inflammatory pathways. Therefore, we studied the effect of KXS on cytokines in the hippocampus and serum of TRD rats.

The effects of KXS on the immune system of TRD rats are concentration-dependent and maximum inhibition of inflammatory factors and promotion of anti-inflammatory cytokines can be obtained by adjusting the dosage of KXS to optimize its antidepressant activity. These results suggest that administration of KXS for fluoxetine-resistant depression in rats was effective in improving depression by influencing the inflammatory processes.
